# A Personal Model of Trumpery: Linguistic Deception Detection in a Real-World High-Stakes Setting

**DOI:** 10.1177/09567976211015941

**Published:** 2021-12-21

**Authors:** Sophie Van Der Zee, Ronald Poppe, Alice Havrileck, Aurélien Baillon

**Affiliations:** 1 Department of Applied Economics, Erasmus School of Economics, Erasmus University Rotterdam; 2 Department of Information and Computing Sciences, Utrecht University; 3 Department of Economics and Management, École Normale Supérieure Paris-Saclay

**Keywords:** deception detection, linguistic analysis, LIWC, Twitter, tailored model, open data, open materials

## Abstract

Language use differs between truthful and deceptive statements, but not all differences are consistent across people and contexts, complicating the identification of deceit in individuals. By relying on fact-checked tweets, we showed in three studies (Study 1: 469 tweets; Study 2: 484 tweets; Study 3: 24 models) how well personalized linguistic deception detection performs by developing the first deception model tailored to an individual: the 45th U.S. president. First, we found substantial linguistic differences between factually correct and factually incorrect tweets. We developed a quantitative model and achieved 73% overall accuracy. Second, we tested out-of-sample prediction and achieved 74% overall accuracy. Third, we compared our personalized model with linguistic models previously reported in the literature. Our model outperformed existing models by 5 percentage points, demonstrating the added value of personalized linguistic analysis in real-world settings. Our results indicate that factually incorrect tweets by the U.S. president are not random mistakes of the sender.

Language use reveals information about who we are and how we feel (Ahmadian et al., 2017). Based on pioneering work in text analysis by Walter Weintraub (1989), methods have been developed to automatically count the types of words people use (Fuller et al., 2006; Pennebaker et al., 2015). These methods make text analysis an efficient and objective approach to study stable traits such as personality (Ahmadian et al., 2017; Weintraub, 1989) and more temporary states such as cooperation (Richardson et al., 2014) and solidarity (Smith et al., 2018). Owing to substantial improvements in automated speech recognition, the applicability of such analyses will only further increase (Hirschberg & Manning, 2015).

Language use also differs between truthful and deceptive statements about past events (Bond & Lee, 2005; Fuller et al., 2015; Newman et al., 2003) and intentions (Kleinberg, van der Toolen, et al., 2018). Linguistic analysis showed that liars tend to experience more cognitive load (e.g., fewer different words and exclusive terms), provide fewer details (e.g., fewer sensory-perceptual words), express more negative emotions (e.g., more anger words), distance themselves more than truth tellers (e.g., fewer first-person pronouns), and refer less to cognitive processes (e.g., fewer insight words; Hauch et al., 2015). However, observed patterns have been partly contradictory and have limited discriminative power (DePaulo et al., 2003). One possible explanation is that the differences between truthful and deceptive language are too small to be consistently observed (Vrij, 2008). Alternatively, there might be significant variation between contexts and individuals in language use, which limits the performance of one-size-fits-all models (Bond et al., 2017). Whereas several researchers have studied the effect of context on cues to deceit (Hauch et al., 2015; Markowitz & Hancock, 2019), the question remains how well the techniques for a whole population can be tailored to a single person. This is important because practitioners must regularly determine whether a specific individual is being deceptive. Answering this question requires a broad set of statements of which the veracity is known, all made by a single individual. To date, this has proven challenging because the fact-checking efforts needed to acquire such a data set are significant.

In the present article, we put language-based lie detection to the test in a unique real-world setting. We analyzed a multitude of statements made by a single individual, the 45th U.S. president. Several organizations have tasked themselves with fact-checking statements of U.S. presidents, including those made in official speeches, Facebook posts, and tweets (Hasen, 2013). Although some messages are posted under the president’s name (e.g., Facebook posts) or are delivered by the president (e.g., speeches), the message itself may have been crafted by other individuals, potentially introducing noise to the signal. Of all fact-checked communication channels, the 45th U.S. president’s Twitter account seemed to be the one he was most in control of (Barbaro, 2015). In addition, tweets are considered official White House communication. A court ruling (Case 1:17-cv-05205-NRB) indeed determined that it was unconstitutional for @realDonaldTrump to block Twitter followers. This official status, combined with systematic fact checking, provides the opportunity for deception detection for a single individual in a high-stakes context.

Previous analyses by independent fact checkers such as *The Washington Post*, *PolitiFact*, and *The Star* concluded that presidential candidates and U.S. presidents regularly make factually incorrect statements (Alterman, 2004; Bond et al., 2017; McGranahan, 2017). These statements are often portrayed as deceptive (Glasser, 2018). However, an incorrect statement does not necessarily imply a lie. The sender may simply be wrong and have a false belief (Bernstein & Loftus, 2009). Being wrong should not affect language use because there is no difference in the perception or intention of the sender. In contrast, when false statements are deliberately presented as truths, one would expect a change in language use, according to the deception hypothesis (Bond & Lee, 2005; Fuller et al., 2015; Hauch et al., 2015). The deception hypothesis postulates that lying can cause behavioral change because lying can be cognitively demanding, elicit emotions and stress, and increase attempted behavioral control (DePaulo et al., 2003; Vrij, 2008; Vrij et al., 2017). We tested the deception hypothesis by investigating whether differences in language can be used to distinguish between factually correct and factually incorrect statements in tweets of the 45th U.S. president.

**Statement of Relevance**

People use different words when they lie and when they tell the truth. Lying is cognitively demanding and can elicit emotional responses and stress, which is reflected in language use. Several studies have identified linguistic differences between truth and lies, but some findings are contradictory. A possible reason is that language use is personal and so is its modification caused by deception. Linguistic analysis at the individual level is therefore a promising approach to lie detection. To test this possibility, we made use of the existence of a unique data set of factually correct and factually incorrect statements of one single individual in a high-stakes setting: the fact-checked tweets of the 45th U.S. president. We developed a personalized language model, which allowed us to predict whether a statement was correct or potentially deceitful with reasonable accuracy. Our personalized model outperformed existing models from the literature, thereby highlighting the added value of an individualized approach.

This research comprised three studies. In Study 1, we analyzed differences in language use between factually correct and factually incorrect tweets and developed a personalized deception-detection model. In Study 2, we tested the out-of-sample performance of our model. In Study 3, we compared the performance of our personalized model with that of previously developed language-based deception-detection models.

## Study 1

### Method

Ethical approval was granted by the internal review board for nonexperimental research of the Erasmus School of Economics, Erasmus University Rotterdam.

#### Data collection

To start, we collected a data set (Data Set 1) of all presidential tweets sent by @realDonaldTrump over the 3-month period from February to April 2018. Data for Study 1 were gathered in May 2018. We examined all tweets posted in the 3 most recent, yet completed, months. The choice of 3 months was a deliberate trade-off between collecting a sufficient number of tweets and the manual labor involved in data screening. In total, 605 tweets were gathered. *The Washington Post* provided us with a data set comprising fact-checked communications from this period that we matched to our Twitter data file. As a result, we could identify which tweets were deemed factually incorrect and labeled the remainder as correct. We named the obtained variable *veracity*. Because fact checking may contain an element of subjectivity, we compared the judged veracity of Data Set 1 with the veracity determined by a second fact-checking source, *PolitiFact* (see the Supplemental Material available online).

#### Data screening

Next, the data set was screened to reduce noise. To facilitate word recognition in linguistic analysis, we corrected misspellings (Bond & Lee, 2005; Newman et al., 2003). Language use reveals information about the sender only when the sender actually writes the text himself or herself, so we removed tweets contaminated by other individuals. We removed 66 retweets and 52 tweets containing quotes of more than six words to eliminate contamination of our data with tweets from other individuals. We also removed 16 duplicate tweets and two tweets solely containing web links, leaving a final data set of 469 tweets with an average tweet length of 33.75 words. Of these 469 tweets, 142 tweets (30.28%) were classified as factually incorrect and 327 as factually correct (69.72%). Some tweets are part of a tweet series. This is indicated by periods at the end of the first tweet and the beginning of succeeding tweets. Although this series of tweets together conveys one message, we did not combine them to avoid artificial inflation of word count. *The Washington Post* fact checked the veracity of these tweets separately, allowing for analysis on the tweet rather than message level.

#### Linguistic cues

We used the 2015 version of the well-validated text-analysis program Linguistic Inquiry and Word Count (LIWC2015; Pennebaker et al., 2015) to compare language use of correct and incorrect tweets. LIWC assigns words to word categories, ranging from standard linguistic dimensions (e.g., personal pronouns, articles, negations) to psychological processes (e.g., positive and negative emotions, cognitive processing), punctuation categories (e.g., periods, commas, exclamation points), formality measures (e.g., swear words, fillers), and meta categories (e.g., analytic, authentic, tone; Pennebaker et al., 2015). Word count is presented as the absolute number of words; all other variables are presented in percentages. Percentages are calculated by counting the number of words belonging to a specific word category and then dividing by word count.

**Fig. 1.**
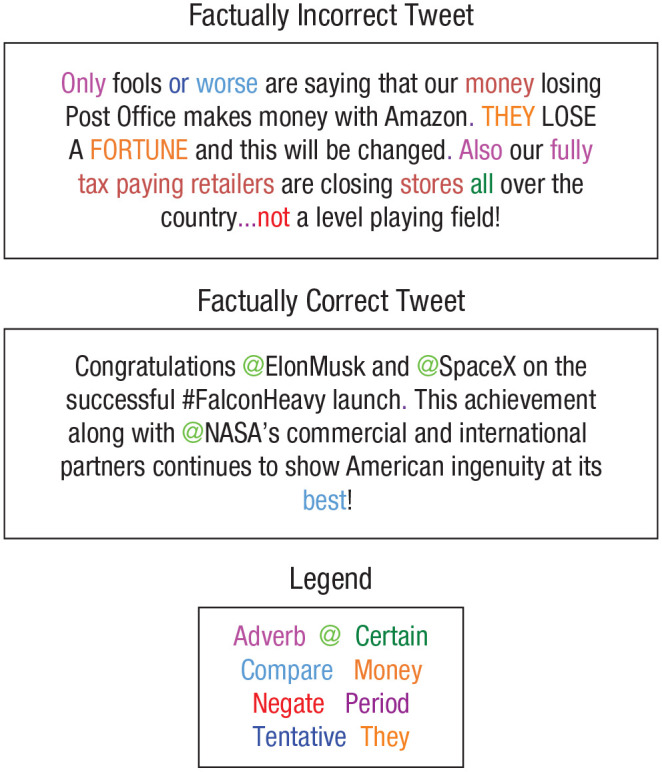
Text of one factually incorrect and one factually correct tweet from Data Set 1 (Study 1). Colors indicate words identified by Linguistic Inquiry and Word Count (LIWC) as belonging to each of nine word categories.

In Study 3, we applied models reported in the literature to our two data sets. However, some of these models from the literature were created using older versions of LIWC (LIWC2001 and LIWC2007), which contained different sets of word categories from LIWC2015. To allow for testing of those older models and to ensure compatibility between models, we used LIWC2015 for the main set of word categories and added relevant word categories from previous LIWC versions. Our final variable set consisted of 103 variables (101 LIWC word categories from three LIWC versions and two additional Twitter variables). Our set contained 92 out of 93 word categories from LIWC2015. The variable *semicolon* was removed because there were no semicolons present in our two data sets. In addition, we added four word categories from LIWC2007 (inclusive, inhibition, exclusive, humans) and five word categories from LIWC2001 (sensory and perceptual processes, optimism, total first person, total third person, metaphysical). Last, we added two variables comprising the percentages of the symbols “@” and “#” present in each tweet because these variables play a central role in Twitter communication patterns but are not included in any LIWC variable. The complete list of included LIWC variables appears in Table S1 in the Supplemental Material. When possible, we have linked LIWC categories to the associated psychological processes on the basis of a categorization by Hauch et al. (2015). In Figure 1, the text of two categorized example tweets is displayed. Colors indicate words identified by LIWC as belonging to each of nine example categories.

### Results

#### Comparing factually correct and incorrect tweets

To test whether language use in factually correct and factually incorrect statements differed, we ran a multivariate analysis of variance (MANOVA). We compared the means of the 101 LIWC variables and two Twitter variables (percentage of @ or # symbols) between the factually correct and the factually incorrect tweets. Using Pillai’s trace, this analysis revealed a main effect of veracity, *V* = .37, *F*(103, 365) = 2.10, *p* < .001 (two-sided, as are all the tests reported in this article), η_*p*_^2^ = .37. Table 1 provides the means for factually correct and incorrect statements in Data Set 1, the *F* statistics, *p* values adjusted with false-discovery-rate (FDR) correction for multiple comparisons (Benjamini & Hochberg, 1995), Bayes factors from a Bayesian *t* test, and Cohen’s *d*s and their 95% confidence intervals. Table 1 includes all variables that were significant at a 5% level with FDR correction (for results for all variables, see Table S1).

**Table 1.** Statistics for Significant Linguistic Inquiry and Word Count (LIWC) Categories in Data Set 1 (Study 1)

LIWC name	Variable name	Psychological process	*M* if correct	*M* if incorrect	*F*(1, 603)	*p*	Bayes factor	Cohen’s *d*
Adverb	Adverbs	Details	3.65	5.36	17.70	.000	503.44	−0.42 [−0.62, −0.22]
Affect	Emotions	Emotion (unspecified)	9.76	7.79	7.15	.026	3.43	0.27 [0.07, 0.47]
Affiliation	Affiliation		3.68	2.30	9.09	.012	8.65	0.30 [0.10, 0.50]
Analytic	Analytic thinking		74.71	68.38	5.76	.048	1.77	0.24 [0.04, 0.44]
Anger	Anger	Emotion (negative)	0.39	0.77	9.64	.011	11.22	−0.31 [−0.51, −0.11]
Apostro	Apostrophes		0.71	1.26	7.04	.026	3.27	−0.27 [−0.46, −0.07]
At	@		1.42	0.11	16.67	.001	310.45	0.41 [0.21, 0.61]
Auxverb	Auxiliary verbs		7.59	9.24	9.45	.012	10.28	−0.31 [−0.51, −0.11]
Cause	Causations	Cognitive load	1.07	1.81	12.25	.004	38.75	−0.35 [−0.55, −0.15]
Certain	Certainty	Certainty	1.80	2.54	6.19	.039	2.18	−0.25 [−0.45, −0.05]
Clout	Clout		70.26	61.17	14.23	.002	98.62	0.38 [0.18, 0.58]
Cogproc	Cognitive processes	Cognitive processes	7.07	10.59	36.86	.000	> 1,000	−0.61 [−0.81, −0.41]
Compare	Comparison words		1.40	2.37	12.07	.004	35.65	−0.35 [−0.55, −0.15]
Differ	Differentiation		1.62	2.63	15.35	.001	167.56	−0.39 [−0.59, −0.19]
Discrep	Discrepancy		1.17	1.73	6.40	.037	2.40	−0.25 [−0.45, −0.06]
Drives	Drives		13.24	10.92	8.40	.016	6.24	0.29 [0.09, 0.49]
Excl	Exclusive	Cognitive load	1.40	2.05	8.18	.016	5.62	−0.29 [−0.49, −0.09]
Exclam	Exclamation marks		5.13	2.81	8.28	.016	5.90	0.29 [0.09, 0.49]
Focuspast	Past orientation		2.48	3.59	10.27	.010	15.19	−0.32 [−0.52, −0.12]
Function	Total function words		43.37	47.58	14.59	.001	116.68	−0.38 [−0.58, −0.18]
Metaph	Metaphysical		1.09	0.16	7.70	.020	4.47	0.28 [0.08, 0.48]
Money	Money		1.26	2.16	8.22	.016	5.71	−0.29 [−0.49, −0.09]
Negate	Negations	Emotion (negative)	1.05	2.51	47.01	.000	> 1,000	−0.69 [−0.89, −0.49]
Negemo	Negative emotions	Emotion (negative)	2.25	3.66	9.99	.010	13.26	−0.32 [−0.52, −0.12]
Other	Total third person	Distance	1.41	2.25	9.88	.010	12.58	−0.32 [−0.51, −0.12]
OtherP	Other punctuation		3.02	1.17	7.21	.026	3.53	0.27 [0.07, 0.47]
Period	Periods		6.24	7.99	9.23	.012	9.24	−0.31 [−0.50, −0.11]
Posemo	Positive emotions	Emotion (positive)	7.43	4.04	23.66	.000	> 1,000	0.49 [0.29, 0.69]
Relig	Religion		0.67	0.05	9.35	.012	9.81	0.31 [0.11, 0.51]
Reward	Reward focus		2.92	1.74	8.47	.016	6.45	0.29 [0.09, 0.49]
Sixltr	Six-letter words	Cognitive load	22.90	20.34	6.32	.037	2.32	0.25 [0.05, 0.45]
Tentat	Tentative	Certainty	1.20	2.16	18.87	.000	872.37	−0.44 [−0.64, −0.24]
They	Third-person plural pronouns		0.73	1.52	16.23	.001	252.35	−0.40 [−0.6, −0.21]
Tone	Emotional tone		67.52	44.73	33.95	.000	> 1,000	0.59 [0.38, 0.79]
Verb	Common verbs		13.43	15.70	9.87	.010	12.56	−0.32 [−0.51, −0.12]
WC	Word count	Cognitive load	31.22	39.57	37.03	.000	> 1,000	−0.61 [−0.81, −0.41]

Note: The *p* values reported here are false-discovery-rate corrected. Bayes factor were obtained from a Bayesian *t* test. Values in brackets after Cohen’s *d*s are 95% confidence intervals. Associated psychological processes are based on categorizations from the study by Hauch et al. (2015).

In total, 36 variables (35.0%) were significant at a 5% level, among which 15 (14.6%) were significant at a 1% level, and 9 (8.7%) were significant at a 0.1% level. These results indicate substantial differences in language use between factually correct and incorrect statements, supporting the deception hypothesis. Correct and incorrect statements differed in terms of cognitive load, certainty, emotions, distance, details, and cognitive processes.

The act of lying can impact language use in different ways. First, lying can be more cognitively demanding than truth telling, leaving fewer cognitive resources for lie construction (DePaulo et al., 2003). As a result, lies are expected to be shorter, less elaborate, and less complex than truths (Hauch et al., 2015). Although the incorrect tweets were indeed less elaborate (i.e., fewer six-letter words), they were also longer (i.e., higher word count) and more complex (i.e., more causations and exclusive words) than truths. Second, liars tend to be more verbally and vocally uncertain than truth tellers (DePaulo et al., 2003), arguably because of a lack of investment or psychological closeness to their deceptive statement. Whereas the incorrect tweets indeed contained more tentative words, they also contained more certainty words. Third, lying can elicit negative emotions (DePaulo et al., 2003). Although incorrect tweets overall contained fewer emotion words than truths, the expressed emotions were more negative. Incorrect tweets contained more anger words and more negative emotion words while containing fewer positive emotion words. Lies also contained more negations, which could indicate a more defensive tone or denial of wrongdoing (Hauch et al., 2015). Fourth, liars tend to be less forthcoming and distance themselves more from their story (DePaulo et al., 2003), resulting in fewer self-references and more other references (Hauch et al., 2015). Incorrect tweets indeed contained fewer first-person pronouns and more third-person pronouns. Fifth, truths and lies typically differ in the type of details. *Reality monitoring* is a verbal-lie-detection tool that relies on differences between actual experiences (i.e., truths) and internally generated events (i.e., lies). In contrast with imagined events, actual experiences are embedded in memory and typically include a variety of perceptual and sensory details (Vrij, 2015). However, we did not find any differences in perceptual details between correct and incorrect tweets and even found an increase rather than a decrease in adverbs. Last, the reality-monitoring approach specifies that liars refer more often to internal, cognitive processes because their story is constructed rather than experienced. We indeed found that incorrect statements contained more cognitive-processing words.

#### Model selection

Next, we aimed to explore whether we can predict the factual correctness or incorrectness of a statement. To do so, we proposed to develop a model to predict factually incorrect statements. We used logit regressions with veracity as the dependent variable and determined which subset of the 103 variables to use as independent variables. Too many variables may lead to overfitting and poor out-of-sample prediction power. Several approaches were possible to select variables for the model. When evaluating these approaches, we considered only the 36 variables that were significant at a 5% level according to the MANOVA because regressions of 469 observations on 103 variables led to perfect separation issues with extreme but nonsignificant coefficients. We compared three different model-selection approaches in detail (see the Supplemental Material). Here, we report the results of the forward-stepwise selection, which gave the most parsimonious model. This approach introduces variables one by one until the Akaike information criterion, which penalizes the log likelihood (measuring the goodness of fit) by the number of variables, does not decrease anymore. We implemented this approach starting with the variable with the highest Cohen’s *d*: negate.

In the remainder of the article, we call the obtained model the *personalized model*. Table 2 reports the marginal effects of the personalized model. For instance, an additional percentage point of negation words increases the chance of an incorrect statement by 2.5 percentage points, whereas a 1-percentage-point increase in religious words decreases the chance of an incorrect statement by 6.7 percentage points. We classified tweets as predicted incorrect if the model assigned a higher chance than we would expect a priori (i.e., higher than 30.28%), and as predicted correct otherwise. We obtained an overall accuracy of 72.92%.

**Table 2.** Marginal Effects of Types of Words and Symbols on the Probability of a Tweet Being Incorrect (Study 1)

LIWC variable	Variable name	Marginal effect	*SE*	*z*	*p*
Negate	Negation	.025	.009	2.925	.003
WC	Word count	.005	.001	3.346	.001
Tone	Emotional tone	−.002	.000	−3.565	.000
At	@	−.050	.019	−2.629	.009
Relig	Religion	−.067	.032	−2.121	.034
Compare	Comparison words	.013	.006	2.183	.029
Period	Periods	.008	.003	2.466	.014
Tentat	Tentative	.015	.008	1.887	.059
Money	Money	.011	.005	2.120	.034
Certain	Certainty	.012	.006	2.013	.044
They	Third-person plural	.016	.009	1.757	.079
Adverb	Adverbs	.011	.005	2.181	.029
Analytic	Analytic thinking	.002	.001	1.809	.070

Note: Variables are expressed as percentage of total words, except word quantity, which is expressed as numbers of words. Marginal effects indicate how much veracity varies when a given explanatory variable varies by one unit, other variables being kept constant at their mean value. Results were obtained with a logit regression (log likelihood = −216.75, *N* = 469).

### Discussion

The deception hypothesis predicts that the type of words people use in truthful and deceptive statements differs. To test the occurrence of language differences, we compared factually correct and incorrect tweets by the U.S. president. Results revealed that 35% (36 of the 104) of the tested word types differed between the two categories, supporting the deception hypothesis. Correct and incorrect tweets differed in terms of cognitive load, certainty, negative emotions, distance, details, and cognitive processes. The sender also used fewer @ symbols, a Twitter function to connect an individual to the sender’s statement. This finding may best be explained by the verifiability approach. An identifiable person who can be traced is an example of a verifiable detail. Liars use fewer verifiable details than truth tellers (Nahari et al., 2014). We also identified differences that were not previously linked to relevant psychological processes, such as punctuation and comparisons. Punctuation can indicate emotions (exclamation marks), suggestions (series of periods, question marks), or the continuation of an idea (periods used in a series of tweets).

Next, we estimated a logit model to determine how accurately we could identify the veracity of individual tweets on the basis of these language cues. The final model contained 13 variables and correctly classified almost three out of four tweets as either factually correct or incorrect solely on the basis of word use. Because the model was built and tested on the same data, overfitting may have occurred, thereby possibly inflating the results. Out-of-sample testing would provide a more accurate estimate of the general performance of our personalized model.

## Study 2

### Method

To test the out-of-sample prediction accuracy of the personalized model developed in Study 1, we collected a second data set (Data Set 2) of presidential tweets. Similar to Data Set 1, Data Set 2 comprised all presidential tweets by @realDonaldTrump covering a 3-month period. Because 3 new months’ worth of tweets was not yet available when we collected Data Set 2, we focused on the tweets in the 3 months preceding the period covered in Data Set 1 (November 2017–January 2018). In total, 606 tweets were gathered. We applied identical screening methods as in Study 1, correcting misspellings and removing 85 retweets, six duplications, two tweets containing severe spelling mistakes, and 29 tweets containing quotes with more than six words. The final data set comprised 484 tweets with an average length of 29.37 words. Of these 484 tweets, 111 (22.93%) were classified as factually incorrect by *The Washington Post*, leaving 373 factually correct tweets (77.07%).

### Results

**Fig. 2.**
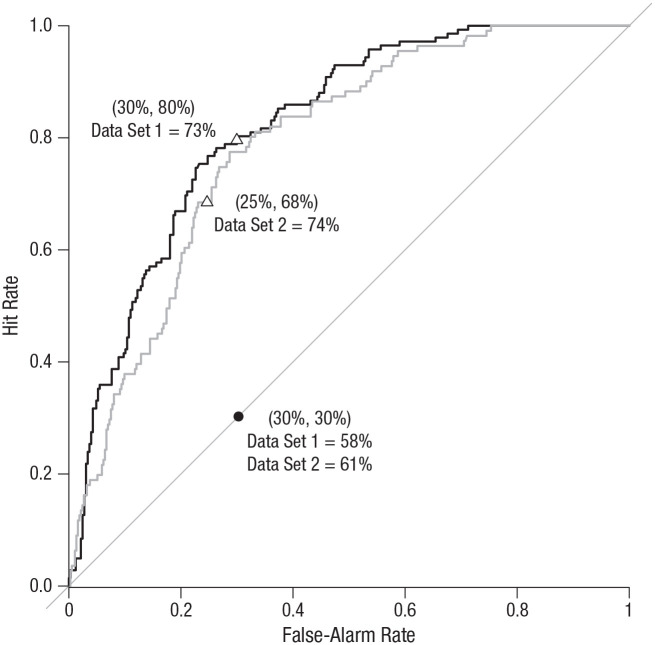
Hit rate as a function of false-alarm rate when the cutoff to classify tweets as predicted correct or predicted incorrect varies (Study 2). Predictions were obtained from the model shown in Table 2. The black curve is associated with Data Set 1 (training set) and the gray curve with Data Set 2 (test set). The diagonal represents random guessing, and the black dot represents the prior used as a random guess. The position of the curve above the diagonal shows the improvement over random guessing. Triangles represent when the prior is used as a cutoff in our model. Coordinates of each of these points (i.e., false-alarm and hit rates) are given in parentheses above the accuracy rate for each data set.

Using the personalized model from Study 1, we computed predicted probabilities on out-of-sample tweets. We used the coefficients obtained on the training set (Data Set 1) and applied them to the variables of the test set (Data Set 2) to predict the probability of each tweet being factually incorrect. This probability was compared with a cutoff (e.g., 50%) to classify tweets as predicted correct or predicted incorrect. A hit occurred when a factually incorrect statement was rightly predicted, and a false alarm occurred when a factually correct statement was wrongly predicted to be incorrect. The resulting receiver operating characteristic (ROC) curves, shown in Figure 2, display hit rates as a function of false-alarm rates when the cutoff varies. The diagonal represents random guessing. The position of the curve above the diagonal shows the improvement over random guessing. It is measured by the area under the curve (AUC); random guessing has an AUC of .5 and a perfect classifier of 1. Figure 2 displays the ROC curves obtained for the test set and for the training set.

The ROC curve for Data Set 2 is lower than the curve for Data Set 1 but still clearly dominates random guessing. The AUC for the test set is .789, compared with .822 for the training set. As could be expected, it decreased but was still very close to .8. To make the results more concrete, one may want to choose a specific cutoff (i.e., to decide which point of the curve to consider) and compute performance metrics for this cutoff. Table 3 reports such metrics. Several approaches to determining the cutoff are possible. The first approach is simply to use .5. In most deception-detection research, a cutoff score of .5 would be a sensible choice because half of the statements in such studies tend to be truthful and half deceptive (Levine, 2018). For the test set, this gives an overall accuracy of 77.69%, a hit rate of 37.84%, and a false-alarm rate of 10.46%, whereas for the training set, overall accuracy is 76.76%, the hit rate is 49.30%, and the false-alarm rate is 11.31%. This threshold is very conservative, missing many incorrect statements, and less applicable because outside the lab, truth–lie base rates are often not 50–50 (Levine, 2018).

**Table 3.** Performance Metrics (Study 2)

Data set and cutoff	Accuracy	95% CI for accuracy	Hit rate	False-alarm rate	Precision	F1	AUC	95% CI for AUC
Data Set 1								
50.00%	76.76%	[72.5, 80.4]	49.30%	11.31%	65.42%	0.562	.822	[.784, .860]
30.28%	72.92%	[70.8, 75.1]	79.58%	29.97%	53.55%	0.640	.822	[.784, .860]
Data Set 2								
50.00%	77.69%	[75.2, 80.0]	37.84%	10.46%	51.85%	0.438	.788	[.745, .832]
30.28%	73.76%	[70.7, 76.7]	68.47%	24.66%	45.24%	0.545	.788	[.745, .832]

Note: Accuracy is defined as the percentage of correct responses. Precision is defined as the number of hits divided by the total number of hits and false alarms. F1 is the harmonic mean of precision and hit rate (also called *recall*). AUC = area under the curve; CI = confidence interval.

An alternative is to rely on the direction of predictions with respect to our prior. In other words, is a tweet more likely to be factually incorrect than we would have expected? To investigate this question, we used the base rate of factually incorrect statements in the training set (30.28%) as the cutoff. We classified statements for which the model gave a higher probability than the base rate as predicted incorrect. This cutoff in the article is intuitive and easy to interpret. In the test set, it gave a hit rate of 68.47% and a false-alarm rate of 24.66%, yielding an overall accuracy of 73.76%, whereas in the training set, it gave a hit rate of 79.58% and a false-alarm rate of 29.96%, yielding an overall accuracy of 72.92%. For comparison, random guessing using the prior probability of 30.28% for any tweet to be incorrect, would obtain an overall accuracy of 60.68%. Alternatively, the more traditional cutoff of 50% would give an accuracy of 50% for random guessing and higher overall accuracy for our model (76.76% for Data Set 1, 77.69% for Data Set 2). Hence, our approach using a prior probability as the cutoff reduces the difference between random guessing and our model, representing a tougher test of our model.

We performed various robustness checks (see the Supplemental Material). We (a) excluded word quantity from the analysis, (b) excluded topical variables such as religion and money, (c) developed models to specific topics (work and money), (d) studied the robustness of the out-of-sample performance to various splits between the training set and test set, and (e) conducted a placebo check. Excluding word quantity of topical variables decreased the out-of-sample accuracy by 4 to 5 percentage points, but the obtained models still clearly outperformed random guessing. Models developed for specific topics led to overfitting, with high within-sample AUCs but much lower out-of-sample ones. The average out-of-sample AUC over 1,000 splits between the training set and test set was .759, whereas the average AUC, if veracity were random (placebo check), was .5 and did not exceed .59 in 1,000 simulations. In summary, our results were not due to chance, and random inaccuracies could not have been predicted with an AUC of .789.

### Discussion

We investigated how consistent the U.S. president’s differences in language use are by predicting the veracity of tweets in an out-of-sample test. We applied the model developed in Study 1 to a new data set to identify classification accuracy on tweets from the same person but different time period. The comparable accuracy within sample (72.92%) and out of sample (73.76%) demonstrates the consistency of the differences in language use over time.

There are several linguistic deception-detection models previously reported in the literature. Our approach is unique because we developed the first personalized model rather than building a model based on statements by multiple people. Whether unique is also meaningful needs further investigation.

## Study 3

### Method

To assess the value of tailoring deception-detection models to a specific individual, we compared the accuracy of our personalized model with models from the literature. Using Google Scholar, we searched for articles containing the terms “deception detection” and “LIWC.” We selected all articles that used LIWC to detect deception and specified the LIWC variables in their models. In total, 24 LIWC-based deception-detection models from 14 articles met our criteria. These models can be divided into three types. *Theoretical* models come from research in which theoretical arguments based on psychological phenomena are made about why some variables should be used in a deception model. For instance, Bond and Lee (2005) developed a LIWC-based deception-detection model based on reality-monitoring principles. Reality monitoring was developed to distinguish true from false memories and relies on differences between such memories, such as sensory and perceptual details, temporal details, emotions, and plausibility. Reality monitoring is now one of the most commonly used methods for verbal deception-detection purposes (Vrij, 2015). As a result, Bond and Lee (2005) included the following LIWC categories in their reality-monitoring-based model: “sensory words,” “spatial words,” “temporal words,” “affective words,” and “cognitive mechanism words” (in LIWC 2015, these categories correspond to “perceptual processes,” “space,” “time,” “affective processes,” and “cognitive processes,” respectively).

By contrast, *data-driven* models have been proposed by authors who, as we did in Study 1, let the data speak. Instead of relying on theory to select variables, data-driven models make selections solely on the basis of their performance in distinguishing between tested conditions (i.e., truthful vs. deceptive).

Finally, *theoretical/data-driven* models are obtained if theoretical models are restricted to the variables that the authors of the corresponding articles found statistically significant. We added a model with only one predictor, word quantity, as an additional benchmark. There are two reasons that this model makes an interesting benchmark. First, veracity can affect statement length, although results in the literature are mixed. In their meta-analysis, Hauch and colleagues (2015) found that liars tend to use fewer words but more sentences. A previous meta-analysis by DePaulo and colleagues (2003) tested several related concepts and found that talking time differed between truth tellers and liars, but response length and duration did not. Second, a longer tweet is simply more likely to contain an inaccuracy. Hence, word quantity may be predictive by itself. By comparing the accuracy of more complex models with the accuracy of this one-variable model, we can see the value of analyzing the content of the tweets beyond their mere length. We also included, as another informative benchmark, random guessing using the prior probability of 30.28%.

For each of the 24 models from the literature, we identified which LIWC variables were included. To allow for a fair comparison between models, instead of relying on coefficients reported in the original articles, we estimated all models on Data Set 1, following the same procedure as for our personalized model in Study 2. We then used the obtained coefficients to compute the accuracy of each model on Data Sets 1 (training) and 2 (test).

### Results

The tested models are shown in Table 4. We report the rank, article, type of data, type of model, model origin, LIWC variables included in the model, and overall accuracy and AUC scores for the training and test sets, respectively. When several models were reported within one article, we differentiated between them in the model column. The models are ranked in terms of overall accuracy on the test set (Data Set 2). Overall accuracy scores on the test set range from 50% to 74%, whereas AUC scores range from .443 to .788. The relatively small differences in classification accuracies of the models on Data Sets 1 and 2 again demonstrate the consistent language use in correct and incorrect tweets.

**Table 4.** Model Comparison (Study 3)

Rank	Article	Context	Communication channel	Model name	Origin	LIWC variable	Training set		Test set	
							Accuracy	AUC	Accuracy	AUC
1	Current article	Political	Twitter	Personalized	Data driven	WC + tone + adverb + negate + compare + tentat + money + relig + period + at + excl	73%	.822	74%	.788
2	Current article	Political	Twitter	Personalized without At	Data driven	WC + tone + adverb + negate + compare + tentat + money + relig + period + excl	73%	.816	72%	.771
3	Ho et al., 2016	Personal experiences	Dyadic chat messages	Full	Theoretical	WC + i + we + you + posemo + negemo + insight + cause + discrep + tentat + certain + inhib + incl + excl + social + negate	68%	.786	69%	.710
4	Matsumoto & Hwang, 2015	Mock crime	Written statements vs. interviews	Backward	Theoretical/data driven	WC + see + posemo + negate + tentat + motion + time	68%	.758	69%	.705
5	Matsumoto & Hwang, 2015	Mock crime	Written statements vs. interviews	Full	Theoretical	WC + i + we + you + shehe + they + see + hear + feel + posemo + negemo + motion + negate + excl + tentat + time	71%	.785	69%	.698
6	Braun et al., 2015	Political	Scripted vs. interactive messages		Theoretical	WC + i + we + shehe + they + negate + negemo	70%	.767	69%	.691
7	Newman et al., 2003	Mock crime Opinions	Videotaped vs. typed vs. handwritten statements	Linguistic dimensions	Theoretical	WC + dic + sixltr + pronoun + i + self + other + negate + article + prep	66%	.741	69%	.677
8	Hancock et al., 2007	Personal experiences	Dyadic chat messages	Full	Theoretical	WC + qmark + WPS + i + you + shehe + they + negemo + negate + excl + cause + senses	70%	.768	69%	.670
9	Toma & Hancock, 2012	Online dating	Profiles	Full	Theoretical/data driven	WC + i + negate + negemo	68%	.754	68%	.677
10	Toma & Hancock, 2012	Online dating	Profiles	Emotional	Theoretical	i + negate + negemo	69%	.726	68%	.672
11	Levitan et al., 2018	Resume	Transcribed interviews		Data driven	WC + achieve + adj + adverb + affiliation + analytic + article + authentic + cause + clout + compare + conj + dash + discrep + drives + family + feel + focusfuture + focuspast + friend + health + interrog + ipron + male + motion + percept + ppron + prep + pronoun + power + relativ + reward + shehe + social + space + swear + verb + we + WPS + you	72%	.800	67%	.688
12	Newman et al., 2003	Mock crime Opinions	Videotaped vs. typed vs. handwritten statements	Psychological processes	Theoretical	affect + posemo + negemo + cogproc + cause + insight + discrep + tentat + certain + senses + social	67%	.734	66%	.696
13	Sharabi & Caughlin, 2019	Dating	Emails		Theoretical	WC + i + excl + negemo	63%	.716	66%	.690
14	Hancock et al., 2007	Personal experiences	Dyadic chat messages	Statistically significant	Theoretical/data driven	WC + i + you + shehe + they + negemo	64%	.725	66%	.681
15	Ho et al., 2016	Personal experiences	Dyadic chat messages	Statistically significant	Theoretical/data driven	WC + we + insight	64%	.686	66%	.665
16	Toma & Hancock, 2012	Online dating	Profiles	Cognitive	Theoretical	WC + excl + motion	62%	.690	65%	.686
17	Kleinberg, Mozes, et al., 2018	Hotel reviews	Written statements	Statistically significant	Data driven	Analytic + dic + function + pronoun + ppron + i + verb + number + allpunc + period + dash + parenth + otherp	60%	.680	65%	.686
18	Bond et al., 2017	Political			Theoretical	percept + space + time + affect + cogproc	65%	.685	64%	.661
19	Word count only					WC	59%	.679	64%	.660
20	Bond & Lee, 2005	Summarizing videos	Transcribed face-to-face interaction	Full	Theoretical	Senses + time + affect + cogproc	64%	.684	64%	.653
21	Newman et al., 2003	Mock crime Opinions	Videotaped vs. typed vs. handwritten statements	Multivariate profile	Theoretical/data driven	i + other + excl + negemo + motion	63%	.671	63%	.652
22	Mihalcea & Strapparava, 2009	Opinions	Transcribed speech		Data driven	Metaph + you + other + humans + certain + optim + i + friend + self + insight	64%	.669	63%	.624
23	Markowitz & Griffin, 2019	Opinions	Written statements		Data driven	Analytic + shehe + auxverb + verb + adj + female + drives + money + nonflu	62%	.658	62%	.637
24	Newman et al., 2003	Mock crime Opinions	Videotaped vs. typed vs. handwritten statements	Relativity	Theoretical	space + incl + excl + motion + time + focuspast + focuspresent + focusfuture	64%	.670	60%	.613
25	Prior					(random guessing with known prior)	58%	.500	61%	.500
26	Kleinberg, Mozes, et al., 2018	Hotel reviews	Written statements	Richness detail	Theoretical	percept + space + time	51%	.545	52%	.526
27	Markowitz & Hancock, 2016	Research articles	Written text		Theoretical	WPS + sixltr + dic	55%	.596	51%	.538
28	Bond & Lee, 2005	Summarizing videos	Transcribed face-to-face interaction	Statistically significant	Theoretical/data driven	Senses + space	52%	.502	50%	.443

Note: The column “LIWC variable” describes the model estimated on the training set. Models are ranked on their accuracy on the test set. LIWC = Linguistic Inquiry and Word Count; AUC = area under the curve.

Our personalized model outperformed all models from the literature by 5 percentage points. This is not surprising on the training set but was not guaranteed on the test set. It shows that we did not overfit the data. Among the best-scoring models from the literature are those that focus on brief chat messages (Ho et al., 2016) or political statements (Braun et al., 2015), both of which consider input that is similar to the political tweets used in the present study. In contrast, the lowest scoring models were developed for other types of data, such as hotel reviews (Kleinberg, Mozes, et al., 2018) and research articles (Markowitz & Hancock, 2016). These results indicate that context matters (Markowitz & Hancock, 2019), even though some models developed for other contexts also performed fairly well. Our model included the variable @, which is specific for tweets. Thus, it might have had an unfair advantage over other models that did not include Twitter-related variables. When we ran our model without the @ variable, the classification accuracy on Data Set 1 remained the same (AUC = .816), whereas the accuracy on Data Set 2 decreased by 2 percentage points (AUC = .771). Despite the minor decrease, this model still outperforms models that have not been developed for a specific individual.

Several variables in our personalized model have not been (e.g., tone, relig) or have rarely been (adverb, compare, money, period) included in other models. This suggests that these variables have not been deemed relevant from a theoretical perspective, nor have they been identified as informative in previous data-driven approaches on other data sets. The superior performance when these terms are present again points to the value of a deception-detection approach tailored to a specific individual in a specific communicative context.

Results also highlight the importance of word count in our data set. The model comprising word count alone achieved a test set accuracy score of 64%. Moreover, nine models in the top 10 included the word-count variable. We did not see a clear relation between classification accuracy and parsimony (i.e., the number of variables included) of a model.

The results on model origin highlight the value of considering psychological processes. Overall, pure theoretical models seem to perform slightly better than theoretical models that are restricted to their significant variables (theoretical/data-driven models). This gives credit to the psychological processes underlying these models, especially when adapted to a context, even though which set of variables best captures these processes may differ from one study to the other.

### Discussion

In Study 3, we investigated how our personalized model performed against models previously reported in the literature. Our personalized model appeared as the best-performing out-of-sample model, in terms of both accuracy and AUC. We attribute this to the inclusion of variables that are specific to the individual under consideration and do not necessarily reflect tendencies of the general population. With our data-driven approach, we could identify these variables, which previously have rarely been investigated.

Together, the way psychological processes are translated in word use seems to differ from one context to the other (Markowitz & Hancock, 2019) and, as the current results show, from one individual to the other. The best-performing models from the literature were developed from theory for contexts (chats, politics) that are comparable with ours. However, restricting the analysis to variables that were significant in the corresponding articles decreased accuracy.

## General Discussion

Previous research demonstrated that people’s language changes when they lie. Most of this research was conducted using single statements from larger groups of people. Here, we investigated whether we could distinguish between correct and incorrect statements made by a single individual. Fact-checked tweets by the 45th U.S. president uniquely allowed for such a comparison. The MANOVA results from Study 1 showed substantial differences in language use between correct and incorrect tweets, suggesting that the sender was in a different cognitive state when constructing factually incorrect tweets. This finding supports the deception hypothesis.

Some of these language differences are in line with previous findings in the deception literature. For example, liars experience more negative emotions (Newman et al., 2003; i.e., increased use of negative emotions and anger words) and higher cognitive load (Vrij et al., 2017; i.e., increased use of cognitive-processing words and decreased use of six-letter words) than truth tellers. Liars also tend to distance themselves more (Toma & Hancock, 2012; i.e., increased use of third-person pronouns and decreased use of first-person pronouns). In contrast with previous findings (Hauch et al., 2015), our results showed that the factually incorrect tweets sent from the @realDonaldTrump account were longer and more complex than the correct ones. Although tweets are restricted in length, we cannot reject the explanation that longer tweets are simply more likely than shorter tweets to contain erroneous statements.

Compared with previous findings on language-based deception, the 73% to 74% overall accuracy of our model for both within-sample and out-of-sample prediction is promising. For example, Bond and colleagues (2017) tested only within-sample accuracy and found classification rates of 63% to 67% based on the communications of U.S. presidential candidates during the 2016 U.S. presidential debates. Sporadically, deception researchers do make out-of-sample predictions, which tend to result in substantial accuracy reductions. For example, Kleinberg, van der Toolen, et al. (2018) reached a within-sample accuracy of 80% in their study on deceptive intentions, but it dropped to 63% in out-of-sample testing. Such accuracy reductions highlight the importance of out-of-sample testing in deception research. Our high out-of-sample accuracy results show that the language differences between true and false statements are stable within the examined individual, providing support for the usefulness of personalized deception models. Improving random guessing by 12 to 15 percentage points when relying on priors and 27 to 28 percentage points when assuming an equal split between truths and lies, this model could help as a screening tool for anyone interested in political fact checking.

A limitation of the current study is that we used the fact-checking output of an independent third party as a proxy for the ground truth, potentially introducing noise in our data. *The Washington Post* labeled statements as incorrect only when they contained incorrect verifiable facts. Opinions, statements that could not be verified, and those about the future are not labeled as factually incorrect, resulting in a conservative estimation of all incorrect tweets.

A second limitation concerns the sender in two manners. First, we examined only one Twitter account. Although Study 3 shows the strength of personalized deception-detection models, the generalizability of such models remains unclear. Future research could test how well this model performs on other types of communication by the U.S. president or tweets by other individuals. Second, not all tweets sent by @realDonaldTrump may have been typed by the president himself (Draper, 2018), introducing noise to the data sets. Journalists have been speculating on how to identify whether the president wrote a tweet himself (Feinberg, 2017), but so far, there is no reliable way to distinguish between writers. A more homogeneous data set comprising tweets solely written by the U.S. president would arguably result in stronger effects.

## Conclusion

Linguistic analysis can test whether incorrect statements are predictable and therefore likely to be deceptive. On the one hand, such models can be a screening tool to help journalists in their work as the fourth pillar of democracy. They could also help in identifying potentially incorrect information on social media, where people increasingly turn for daily news (Allcott & Gentzkow, 2017). On the other hand, any person having access to people’s personal posts and a way to approximate ground truth could develop a model such as the one in this article. Therefore, these results also constitute a warning to all individuals posting a wealth of private, or not so private, information online.

## Supplemental Material

sj-docx-1-pss-10.1177_09567976211015941 – Supplemental material for A Personal Model of Trumpery: Linguistic Deception Detection in a Real-World High-Stakes Setting Supplemental material, sj-docx-1-pss-10.1177_09567976211015941 for A Personal Model of Trumpery: Linguistic Deception Detection in a Real-World High-Stakes Setting by Sophie Van Der Zee, Ronald Poppe, Alice Havrileck and Aurélien Baillon in Psychological Science

## Transparency

*Action Editor:* Vladimir Sloutsky

*Editor:* Patricia J. Bauer

*Author Contributions*

S. Van Der Zee conceived the idea for this research and gathered the data. S. Van Der Zee and A. Havrileck screened the data and prepared it for analysis. All the authors analyzed the data. The figures were created for the article and for conference presentations by R. Poppe and A. Baillon, and the website for the article was created by A. Havrileck. S. Van Der Zee, R. Poppe, and A. Baillon wrote the original draft of the manuscript, and all the authors provided feedback and approved the final manuscript for submission.

**Open Practices:** All data and analysis code have been made publicly available via Figshare and can be accessed at https://doi.org/10.25397/eur.17179514. The design and analysis plans for the studies were not preregistered. This article has received the badges for Open Data and Open Materials. More information about the Open Practices badges can be found at http://www.psychologicalscience.org/publications/badges.
