# Supplementary material for: A Personal Model of Trumpery: Linguistic Deception Detection in a Real-World High-Stakes Setting
Source: Psychol Sci. 2021 Dec 21;33(1):3–17. doi: 10.1177/09567976211015941 (PMC13033364; doi:10.1177/09567976211015941)
Supplement: sj-docx-1-pss-10.1177_09567976211015941 – Supplemental material for A Personal Model of Trumpery: Linguistic Deception Detection in a Real-World High-Stakes Setting [file sj-docx-1-pss-10.1177_09567976211015941.docx]

Supplemental material for
“A personal model of trumpery:
 Linguistic deception detection in a real-world high-stakes setting”

# Fact-check comparison between Washington Post and PolitiFact

Dataset 1 comprises 605 tweets. After data screening, 469 tweets remained. Of these, the Washington Post judged 142 tweets as factually incorrect. In this paper, fact-checking by the Washington Post is used as a proxy of ground truth. However, fact-checking may contain an element of subjectivity, possibly affecting veracity judgements. To test the level of subjectivity, we collected veracity judgements for Dataset 1 by a second independent fact-checker, PolitiFact, and compared their judgements to those of the Washington Post. Fifteen tweets from Dataset 1 were fact-checked by PolitiFact. PolitiFact judges communications to be either True, Mostly True, Half True, Mostly False, False, or Pants on Fire. When labeling True, Mostly True, and Half True judgements as ‘factually correct’, and Mostly False, False, and Pants on Fire judgements as ‘factually incorrect’, veracity judgements of 12 out of 15 tweets matched (80.0%). Two tweets were judged as Half True by PolitiFact. Since Half True also indicates Half False, one could argue that the PolitiFact judgement Half True matches both the Washington Post’s correct and incorrect judgement. In this case, 13 out of 15 tweets match (86.7%). Together, these findings indicate that a) the two independent fact-checkers largely agree, and b) the Washington Post fact-checked more systematically than PolitiFact. These results strengthen the argument for relying on veracity judgements by the Washington Post for this paper.

# Statistics for All LIWC Categories

Table 1 in the main text reports statistics for significant LIWC categories only. Table S1 reports the same statistics for all LIWC categories.

Table S1 *Statistics for the LIWC categories*

| LIWC name | Variable name | Psychological process | Mean if correct | Mean if incorrect | F stat. | p-value | Sig. | Bayes Factor | Cohen's d | CI |
| --- | --- | --- | --- | --- | --- | --- | --- | --- | --- | --- |
| achieve | Achievement |  | 2.23 | 1.59 | 4.80 | 0.073 |  | 1.12 | 0.22 | [0.02,0.42] |
| adj | Adjectives | Details | 6.45 | 6.16 | 0.21 | 0.764 |  | 0.12 | 0.05 | [-0.15,0.24] |
| adverb | Adverbs | Details | 3.65 | 5.36 | 17.70 | 0.000 | *** | 503.44 | -0.42 | [-0.62,-0.22] |
| affect | Emotions | Emotion (unspecified) | 9.76 | 7.79 | 7.15 | 0.026 | * | 3.43 | 0.27 | [0.07,0.47] |
| affiliation | Affiliation |  | 3.68 | 2.30 | 9.09 | 0.012 | * | 8.65 | 0.30 | [0.10,0.50] |
| AllPunc | All punctuation |  | 17.60 | 16.11 | 1.53 | 0.349 |  | 0.23 | 0.12 | [-0.07,0.32] |
| Analytic | Analytic thinking |  | 74.71 | 68.38 | 5.76 | 0.048 | * | 1.77 | 0.24 | [0.04,0.44] |
| anger | Anger | Emotion (negative) | 0.39 | 0.77 | 9.64 | 0.011 | * | 11.22 | -0.31 | [-0.51,-0.11] |
| anx | Anxiety | Emotion (negative) | 0.12 | 0.25 | 3.26 | 0.144 |  | 0.54 | -0.18 | [-0.38,0.02] |
| Apostro | Apostrophes |  | 0.71 | 1.26 | 7.04 | 0.026 | * | 3.27 | -0.27 | [-0.46,-0.07] |
| article | Articles |  | 6.43 | 6.65 | 0.27 | 0.745 |  | 0.13 | -0.05 | [-0.25,0.15] |
| assent | Assent |  | 0.10 | 0.04 | 0.36 | 0.707 |  | 0.13 | 0.06 | [-0.14,0.26] |
| At | @ |  | 1.42 | 0.11 | 16.67 | 0.001 | *** | 310.45 | 0.41 | [0.21,0.61] |
| Authentic | Authentic |  | 32.69 | 29.89 | 0.77 | 0.562 |  | 0.16 | 0.09 | [-0.11,0.29] |
| auxverb | Auxiliary verbs |  | 7.59 | 9.24 | 9.45 | 0.012 | * | 10.28 | -0.31 | [-0.51,-0.11] |
| bio | Biology |  | 0.86 | 0.56 | 2.38 | 0.216 |  | 0.35 | 0.15 | [-0.04,0.35] |
| body | Body |  | 0.13 | 0.07 | 0.59 | 0.607 |  | 0.15 | 0.08 | [-0.12,0.27] |
| cause | Causations | Cognitive load | 1.07 | 1.81 | 12.25 | 0.004 | ** | 38.75 | -0.35 | [-0.55,-0.15] |
| certain | Certainty | Certainty | 1.80 | 2.54 | 6.19 | 0.039 | * | 2.18 | -0.25 | [-0.45,-0.05] |
| Clout | Clout |  | 70.26 | 61.17 | 14.23 | 0.002 | ** | 98.62 | 0.38 | [0.18,0.58] |
| cogproc | Cognitive processes | Cognitive processes | 7.07 | 10.59 | 36.86 | 0.000 | *** | >1,000 | -0.61 | [-0.81,-0.41] |
| Colon | Colons |  | 0.40 | 0.07 | 5.50 | 0.054 |  | 1.57 | 0.24 | [0.04,0.43] |
| Comma | Commas |  | 0.01 | 0.03 | 0.74 | 0.564 |  | 0.16 | -0.09 | [-0.28,0.11] |
| compare | Comparison words |  | 1.40 | 2.37 | 12.07 | 0.004 | ** | 35.65 | -0.35 | [-0.55,-0.15] |
| conj | Conjunctions |  | 4.76 | 5.00 | 0.40 | 0.686 |  | 0.14 | -0.06 | [-0.26,0.13] |
| Dash | Dashes |  | 0.65 | 0.58 | 0.14 | 0.824 |  | 0.12 | 0.04 | [-0.16,0.23] |
| death | Death |  | 0.38 | 0.16 | 2.29 | 0.221 |  | 0.33 | 0.15 | [-0.05,0.35] |
| Dic | Dictionary words |  | 79.23 | 81.26 | 3.26 | 0.144 |  | 0.54 | -0.18 | [-0.38,0.02] |
| differ | Differentiation |  | 1.62 | 2.63 | 15.35 | 0.001 | ** | 167.56 | -0.39 | [-0.59,-0.19] |
| discrep | Discrepancy |  | 1.17 | 1.73 | 6.40 | 0.037 | * | 2.40 | -0.25 | [-0.45,-0.06] |
| drives | Drives |  | 13.24 | 10.92 | 8.40 | 0.016 | * | 6.24 | 0.29 | [0.09,0.49] |
| excl | Exclusive | Cognitive load | 1.40 | 2.05 | 8.18 | 0.016 | * | 5.62 | -0.29 | [-0.49,-0.09] |
| Exclam | Exclamation marks |  | 5.13 | 2.81 | 8.28 | 0.016 | * | 5.90 | 0.29 | [0.09,0.49] |
| family | Family |  | 0.23 | 0.06 | 4.45 | 0.085 |  | 0.95 | 0.21 | [0.01,0.41] |
| feel | Feeling | Details | 0.39 | 0.16 | 3.43 | 0.136 |  | 0.58 | 0.19 | [-0.01,0.38] |
| female | Female references |  | 0.47 | 0.50 | 0.01 | 0.962 |  | 0.11 | -0.01 | [-0.21,0.19] |
| filler | Fillers |  | 0.01 | 0.00 | 0.43 | 0.674 |  | 0.14 | 0.07 | [-0.13,0.26] |
| focusfuture | Future orientation |  | 1.79 | 1.22 | 4.94 | 0.071 |  | 1.19 | 0.22 | [0.03,0.42] |
| focuspast | Past orientation |  | 2.48 | 3.59 | 10.27 | 0.010 | ** | 15.19 | -0.32 | [-0.52,-0.12] |
| focuspresent | Present orientation |  | 8.65 | 10.10 | 5.41 | 0.056 |  | 1.49 | -0.23 | [-0.43,-0.04] |
| friend | Friends |  | 0.19 | 0.15 | 0.28 | 0.745 |  | 0.13 | 0.05 | [-0.14,0.25] |
| function. | Total function words |  | 43.37 | 47.58 | 14.59 | 0.001 | ** | 116.68 | -0.38 | [-0.58,-0.18] |
| Hashtag | # |  | 1.19 | 0.07 | 3.81 | 0.113 |  | 0.70 | 0.20 | [0.00,0.39] |
| health | Health |  | 0.38 | 0.41 | 0.03 | 0.907 |  | 0.11 | -0.02 | [-0.22,0.18] |
| hear | Hearing | Details | 0.32 | 0.41 | 0.68 | 0.585 |  | 0.15 | -0.08 | [-0.28,0.11] |
| home | Home |  | 0.45 | 0.26 | 2.97 | 0.160 |  | 0.47 | 0.17 | [-0.02,0.37] |
| humans | Humans |  | 0.92 | 0.77 | 0.62 | 0.601 |  | 0.15 | 0.08 | [-0.12,0.28] |
| i | First-person singular pronouns | Distance | 1.23 | 1.13 | 0.21 | 0.764 |  | 0.12 | 0.05 | [-0.15,0.24] |
| incl | Inclusive |  | 5.82 | 5.04 | 2.92 | 0.162 |  | 0.45 | 0.17 | [-0.03,0.37] |
| informal | Informal |  | 0.27 | 0.23 | 0.11 | 0.843 |  | 0.12 | 0.03 | [-0.16,0.23] |
| ingest | Ingestion |  | 0.16 | 0.07 | 1.13 | 0.450 |  | 0.19 | 0.11 | [-0.09,0.30] |
| inhib | Inhibition |  | 0.77 | 1.18 | 3.84 | 0.113 |  | 0.71 | -0.20 | [-0.39,0.00] |
| insight | Insight | Cognitive processes | 1.02 | 1.11 | 0.23 | 0.764 |  | 0.12 | -0.05 | [-0.25,0.15] |
| interrog | Interrogatives |  | 0.91 | 1.34 | 4.74 | 0.073 |  | 1.09 | -0.22 | [-0.42,-0.02] |
| ipron | Impersonal pronouns |  | 2.94 | 3.20 | 0.67 | 0.585 |  | 0.15 | -0.08 | [-0.28,0.12] |
| leisure | Leisure |  | 0.64 | 0.32 | 2.85 | 0.166 |  | 0.44 | 0.17 | [-0.03,0.37] |
| male | Male references |  | 0.83 | 0.77 | 0.06 | 0.886 |  | 0.11 | 0.02 | [-0.17,0.22] |
| Metaph | Metaphysical |  | 1.09 | 0.16 | 7.70 | 0.020 | * | 4.47 | 0.28 | [0.08,0.48] |
| money | Money |  | 1.26 | 2.16 | 8.22 | 0.016 | * | 5.71 | -0.29 | [-0.49,-0.09] |
| motion | Motion | Details | 1.64 | 1.51 | 0.26 | 0.745 |  | 0.13 | 0.05 | [-0.15,0.25] |
| negate | Negations | Emotion (negative) | 1.05 | 2.51 | 47.01 | 0.000 | *** | >1,000 | -0.69 | [-0.89,-0.49] |
| negemo | Negative emotions | Emotion (negative) | 2.25 | 3.66 | 9.99 | 0.010 | * | 13.26 | -0.32 | [-0.52,-0.12] |
| netspeak | Netspeak |  | 0.06 | 0.11 | 0.92 | 0.504 |  | 0.17 | -0.10 | [-0.29,0.10] |
| nonflu | Nonfluencies |  | 0.08 | 0.07 | 0.04 | 0.896 |  | 0.11 | 0.02 | [-0.18,0.22] |
| number | Numbers | Details | 1.58 | 1.92 | 1.00 | 0.482 |  | 0.18 | -0.10 | [-0.30,0.10] |
| Optim | Optimism |  | 1.08 | 1.03 | 0.08 | 0.886 |  | 0.12 | 0.03 | [-0.17,0.23] |
| Other | Total third person | Distance | 1.41 | 2.25 | 9.88 | 0.010 | * | 12.58 | -0.32 | [-0.51,-0.12] |
| OtherP | Other punctuation |  | 3.02 | 1.17 | 7.21 | 0.026 | * | 3.53 | 0.27 | [0.07,0.47] |
| Parenth | Parentheses (pairs) |  | 0.62 | 1.09 | 3.87 | 0.113 |  | 0.72 | -0.20 | [-0.40,0.00] |
| percept | Perceptual processes |  | 1.59 | 1.31 | 1.06 | 0.466 |  | 0.19 | 0.10 | [-0.09,0.30] |
| Period | Periods |  | 6.24 | 7.99 | 9.23 | 0.012 | * | 9.24 | -0.31 | [-0.50,-0.11] |
| posemo | Positive emotions | Emotion (positive) | 7.43 | 4.04 | 23.66 | 0.000 | *** | >1,000 | 0.49 | [0.29,0.69] |
| power | Power |  | 5.09 | 5.27 | 0.14 | 0.824 |  | 0.12 | -0.04 | [-0.23,0.16] |
| ppron | Personal pronouns |  | 6.03 | 5.62 | 0.56 | 0.617 |  | 0.15 | 0.08 | [-0.12,0.27] |
| prep | Prepositions | Details | 12.7 | 12.83 | 0.06 | 0.886 |  | 0.11 | -0.02 | [-0.22,0.17] |
| pronoun | Total pronouns | Distance | 8.96 | 8.82 | 0.05 | 0.886 |  | 0.11 | 0.02 | [-0.17,0.22] |
| QMark | Question marks |  | 0.44 | 0.44 | 0.00 | 0.987 |  | 0.11 | 0.00 | [-0.20,0.20] |
| quant | Quantifiers | Details | 1.91 | 2.31 | 1.65 | 0.327 |  | 0.25 | -0.13 | [-0.33,0.07] |
| Quote | Quotation marks |  | 0.37 | 0.66 | 3.22 | 0.144 |  | 0.52 | -0.18 | [-0.38,0.02] |
| relativ | Relativity |  | 14.38 | 13.79 | 0.46 | 0.665 |  | 0.14 | 0.07 | [-0.13,0.27] |
| relig | Religion |  | 0.67 | 0.05 | 9.35 | 0.012 | * | 9.81 | 0.31 | [0.11,0.51] |
| reward | Reward focus |  | 2.92 | 1.74 | 8.47 | 0.016 | * | 6.45 | 0.29 | [0.09,0.49] |
| risk | Risk focus |  | 0.87 | 1.35 | 4.84 | 0.073 |  | 1.14 | -0.22 | [-0.42,-0.02] |
| sad | Sadness | Emotion (negative) | 0.49 | 0.87 | 4.41 | 0.085 |  | 0.93 | -0.21 | [-0.41,-0.01] |
| see | Seeing | Details | 0.86 | 0.73 | 0.34 | 0.714 |  | 0.13 | 0.06 | [-0.14,0.26] |
| Self | Total first person | Distance | 3.54 | 2.78 | 3.59 | 0.126 |  | 0.62 | 0.19 | [-0.01,0.39] |
| Senses | Sensory and Perceptual Processes | Details | 1.14 | 1.16 | 0.01 | 0.962 |  | 0.11 | -0.01 | [-0.21,0.19] |
| sexual | Sexual |  | 0.03 | 0.00 | 1.16 | 0.446 |  | 0.19 | 0.11 | [-0.09,0.31] |
| shehe | Third-person singular pronouns |  | 0.68 | 0.73 | 0.06 | 0.886 |  | 0.11 | -0.02 | [-0.22,0.17] |
| Sixltr | Six-letter words | Cognitive load | 22.9 | 20.34 | 6.32 | 0.037 | * | 2.32 | 0.25 | [0.05,0.45] |
| social | Social processes |  | 9.37 | 8.32 | 2.35 | 0.216 |  | 0.35 | 0.15 | [-0.04,0.35] |
| space | Space | Details | 8.14 | 8.10 | 0.00 | 0.966 |  | 0.11 | 0.01 | [-0.19,0.20] |
| swear | Swear words |  | 0.01 | 0.01 | 0.01 | 0.962 |  | 0.11 | -0.01 | [-0.20,0.19] |
| tentat | Tentative | Certainty | 1.20 | 2.16 | 18.87 | 0.000 | *** | 872.37 | -0.44 | [-0.64,-0.24] |
| they | Third-person plural pronouns |  | 0.73 | 1.52 | 16.23 | 0.001 | *** | 252.35 | -0.4 | [-0.60,-0.21] |
| time | Time | Details | 5.12 | 4.43 | 1.81 | 0.297 |  | 0.27 | 0.14 | [-0.06,0.33] |
| Tone | Emotional tone |  | 67.52 | 44.73 | 33.95 | 0.000 | *** | >1,000 | 0.59 | [0.38,0.79] |
| verb | Common verbs |  | 13.43 | 15.70 | 9.87 | 0.010 | * | 12.56 | -0.32 | [-0.51,-0.12] |
| WC | Word quantity | Cognitive load | 31.22 | 39.57 | 37.03 | 0.000 | *** | >1,000 | -0.61 | [-0.81,-0.41] |
| we | First-person plural pronouns | Distance | 2.31 | 1.65 | 3.12 | 0.148 |  | 0.50 | 0.18 | [-0.02,0.38] |
| work | Job/Work |  | 5.08 | 5.12 | 0.01 | 0.962 |  | 0.11 | -0.01 | [-0.20,0.19] |
| WPS | Average sentence length | Cognitive load | 12.87 | 14.09 | 3.20 | 0.144 |  | 0.52 | -0.18 | [-0.38,0.02] |
| you | Total second-person pronouns | Distance | 1.07 | 0.59 | 2.44 | 0.211 |  | 0.36 | 0.16 | [-0.04,0.35] |

*Note.* Means for factual correct and incorrect statements in Dataset 1 are presented. Column 6 reports F-statistics and column 7 p-values adjusted with False Detection Rate (FDR) correction using the method of Benjamini & Hochberg (1995). Significance is reported in column 8 on three levels: *: p<0.05; **: p<0.01; ***: p<0.001. Column 9 reports the Bayes factor of a Bayes t-test and column 10 Cohen’s d and its 95% confidence interval. Associated psychological processes are based on categorizations from Hauch et al. (2015).

# Model Selection

In the main text, we build the personalized model using forward-stepwise variable selection. Several alternative approaches were possible, which we compare here. When evaluating these approaches, we only considered the 36 variables that were significant at a 5% level (after FDR correction) according to the MANOVA because regressions of 469 observations on 103 variables led to perfect separation issues with extreme but non-significant coefficients.

We took three different model selection approaches. First, we used a stepwise selection of variables, either forward or backward, using AIC as a criterion to stop the process. The forward-stepwise selection (model “Forward”) means introducing variables one by one until the AIC does not decrease anymore. We implemented this approach starting with the variable with the highest (absolute) Cohen’s d: Negation. The backward-stepwise approach (model “Backward”) starts with the whole set of 36 variables and removes them one by one, until the AIC does not decrease anymore, an approach previously used by Matsumoto & Hwang (2015). Second, we also followed the approaches of (Bond & Lee, 2005; Fuller et al., 2015; Newman et al., 2003; Bond et al., 2017) and used the variables that were significant in the MANOVA. This gave us two possible models. Model “36 variables” includes the variables that were significant at 5% in the MANOVA and model “15 variables” includes the variables that were significant at 1% in the MANOVA. Third, Least Absolute Shrinkage and Selection Operator (LASSO) directly penalizes the presence of variables in the regression. We applied this approach and selected variables by LASSO (model “Lasso”) with the penalty level (known as lambda) that gives the lowest cross-validation mean standard errors. We evaluated the different models on Dataset 1. The obtained results are presented in Table S2.

Table S2 *Exploration of common variable selection methods*

| Model | Number of Variables | Log-likelihood | AIC | AUC |
| --- | --- | --- | --- | --- |
| Forward | 13 | -216.75 | 461.50 | 0.822 |
| Backward | 13 | -216.75 | 461.50 | 0.822 |
| 36 variables | 36 | -209.99 | 493.98 | 0.839 |
| 15 variables | 15 | -226.02 | 484.03 | 0.805 |
| Lasso | 19 | -215.04 | 470.07 | 0.826 |

*Note.* Number of variables, log-likelihood, AIC, and AUC scores are reported for each model on Dataset 1.

The Forward and Backward selection procedures gave the same model, with 13 variables. As could be expected, the model with all 36 variables has the highest log-likelihood. In terms of AIC, models Forward and Backward score better (i.e., get lower AIC) than the other models, which is not surprising because they were developed using AIC as selection criterion. More interestingly, in terms of AUC, all models perform closely from each other, with values 0.82-0.84 meaning that they are all closer to perfect classification than to random guessing. To conclude, the Forward (or equivalently Backward) model manages to obtain comparable results than the other models in terms of AUC but with much more parsimony, using only 13 variables. This is the model implemented and reported in the paper, under the name *personalized model*.

# Robustness Check 1: Excluding WC

We replicated Study 1 and Study 2 after excluding word quantity (WC). The MANOVA results remain qualitatively unchanged: main effect of veracity *V* = .37, *F* (102, 366) = 2.08, *p* < 0.001 (two-sided, as all the tests reported in this paper), η^2^_p_ = .37.

In the model selection, the Backward model reaches a slightly lower AIC than the Forward model but performs slightly worse in terms of AUC. For comparability with the main results, we ran the rest of the analysis with the Forward model. When comparing Table S2 to Table S3, we see that removing WC reduces the log-likelihood but the AUC remains almost the same, except for the model with the 13 variables that were significant at a one percent level.

Table S3 *Exploration of common variable selection methods without WC*

| Model | Number of Variables | Log-likelihood | AIC | AUC |
| --- | --- | --- | --- | --- |
| Forward | 14 | -218.52 | 467.05 | 0.816 |
| Backward | 15 | -217.20 | 466.39 | 0.822 |
| 35 variables | 35 | -212.32 | 496.63 | 0.835 |
| 13 variables | 13 | -231.43 | 490.87 | 0.798 |
| Lasso | 24 | -213.41 | 476.82 | 0.833 |

We display in Table S4 the marginal effect of the Forward model, to be compared with Table 2 in the paper. Nine variables of the personalized model are still part of the model, (rows in bold) with very similar coefficients. Five variables are in the new model that were not in the personalized model with WC. Only two of them are significant: Exclamation Marks and Six-letter Words. A higher proportion of exclamation marks and six-letter words is associated with a higher probability for the tweet being factually correct. It is likely to be an artifact of not correcting for word quantity. From the model reported in the main text, the final three variables (Third-person plural, Adverbs, Analytic thinking), i.e. the variables that were selected last in the forward-stepwise selection (two of them being not significant) are not present anymore in the model without WC.

Table S4 *Marginal effects of types of words/symbols on the probability of a tweet being incorrect*

| LIWC variables | Variable name | Marginal effects | Standard errors | *z*-score | *p*-value |
| --- | --- | --- | --- | --- | --- |
| **negate** | **Negation** | **.028** | **.009** | **3.034** | **.002** |
| **Tone** | **Emotional Tone** | **-.002** | **.000** | **-3.690** | **.000** |
| **At** | **@** | **-.048** | **.019** | **-2.534** | **.011** |
| Exclam | Exclamation marks | -.012 | .005 | -2.349 | .019 |
| **relig** | **Religion** | **-.062** | **.031** | **-1.994** | **.046** |
| **compare** | **Comparison words** | **.015** | **.006** | **2.433** | **.015** |
| Sixltr | Six-letter words | -.005 | .002 | -2.051 | .040 |
| **money** | **Money** | **.013** | **.005** | **2.405** | **.016** |
| **certain** | **Certainty** | **.014** | **.007** | **2.165** | **.030** |
| **tentat** | **Tentative** | **.020** | **.008** | **2.425** | **.015** |
| **Period** | **Periods** | **.006** | **.003** | **1.688** | **.091** |
| Cause | Causation | .013 | .009 | 1.547 | .122 |
| Excl | Exclusive | -.014 | .010 | -1.456 | .145 |
| affiliation | Affiliation | -.007 | .005 | -1.426 | .154 |

*Note*. Rows in bold correspond to the variables that are also in the personalized model.

Replicating Study 2, we obtained an out-of-sample accuracy on Dataset 2 of 69.01% and an AUC of 0.765, both a few points lower than in the analysis reported in the main text. Since the AUC on the train set without WC is comparable to what we obtained when we included WC, it seems that excluding WC induced overfitting, hurting the performance of the obtained model on the test set.

# Robustness Check 2: Excluding Topical Variables

If a topical variable predicts that the tweet is factual incorrect, it is uncertain whether it comes from the sender’s lack of knowledge in this topic or from the sender being more deceiving about this topic. Hence, we also replicated Studies 1 and 2 after excluding topical variables, namely Achievement, Biology, Body, Death, Health, Home, Ingestion, Leisure, Metaphysical, Money, Religion, Sexual, and Work. Again, the MANOVA results remain comparable: main effect of veracity *V* = .35, *F* (90, 378) = 2.31, *p* < 0.001 (two-sided, as all the tests reported in this paper), η^2^_p_ = .35. A total of 33 variables are significant at a 5% level (Metaphysical, Money and Religion that were significant at the 5% level in Study 1 are now excluded because they are topical variables).

Table S5 *Exploration of common variable selection methods without topical variables*

| Model | Number of Variables | Log-likelihood | AIC | AUC |
| --- | --- | --- | --- | --- |
| Forward | 10 | -224.16 | 470.32 | 0.809 |
| Backward | 14 | -220.23 | 470.46 | 0.816 |
| 33 variables | 33 | -216.07 | 500.15 | 0.827 |
| 19 variables | 19 | -224.54 | 489.07 | 0.809 |
| Lasso | 16 | -221.55 | 477.1 | 0.814 |

The Forward model with 10 variables is the most parsimonious and reaches the lowest AIC. Nine of these variables are the same as in the personalized model reported in the main text. Besides Money and Religion, Adverbs and Affiliation are not present anymore in the model. Instead, Affiliation is. As in the previous robustness check, it is worth noting that the variables selected last are not always the same, but the main ones (Negation, Emotional Tone, @, Comparison Words) remain.

Table S6 *Marginal effects of types of words/symbols on the probability of a tweet being incorrect*

| LIWC variables | Variable name | Marginal effects | Standard errors | *z*-score | *p*-value |
| --- | --- | --- | --- | --- | --- |
| **negate** | **Negation** | .027 | .009 | 3.122 | .002 |
| **WC** | **Word Quantity** | .006 | .002 | 3.634 | .000 |
| **Tone** | **Emotional Tone** | -.002 | .000 | -3.825 | .000 |
| **At** | **@** | -.058 | .020 | -2.871 | .004 |
| **compare** | **Comparison Words** | .016 | .006 | 2.543 | .011 |
| **Period** | **Periods** | .008 | .003 | 2.366 | .018 |
| **tentat** | **Tentative** | .016 | .008 | 1.904 | .057 |
| **certain** | **Certainty** | .012 | .006 | 1.844 | .065 |
| affiliation | Affiliation | -.009 | .005 | -1.624 | .104 |
| **they** | **Third-person plural** | .014 | .009 | 1.508 | .131 |

*Note*. Rows in bold correspond to the variables that are also in the personalized model.

Replicating Study 2, we found that the model described in the previous table gives an accuracy on Dataset 2 of 70.66% and an AUC of 0.754, both slightly lower than the model with Money and Religion.

# Robustness Check 3: Topic-Specific Models

We also tried to derive topic-specific models. We first made two subsets of Dataset 1 and two subsets of Dataset 2 by separating tweets with at least one money-related word (i.e., those for which the variable Money was not 0) from those without (Money = 0). We obtained 4 datasets: Dataset 1_Money (*N* = 138), Dataset 1_NoMoney (*N* = 331), Dataset 2_Money (*N* = 179), Dataset 2_NoMoney (*N* = 305). We could not replicate the MANOVA because there were too few tweets for too many variables. We could, however, obtain a model with the same Forward approach as we used in the main text (starting with WC and using the same maximal set of 36 variables). We obtained such a model on Dataset 1_Money and another one on Dataset 1_NoMoney. We then used these two models to make out-of-sample predictions on Dataset 2_Money and Dataset 2_NoMoney, respectively.

We conducted the same analysis by splitting the dataset according to the Work variable, giving Dataset 1_Work (*N* = 340), Dataset 1_NoWork (*N* = 129), Dataset 2_Work (*N* = 310), Dataset 2_NoWork (*N* = 174). Work and Money were the only variables such that all sub-datasets would have at least 100 tweets.

Table S7 *AUC of models restricted to specific topics*

| AUC | Money | No Money | Work | No Work |
| --- | --- | --- | --- | --- |
| Train set (Dataset 1) | 0.829 | 0.823 | 0.820 | 0.929 |
| Test set (Dataset 2) | 0.606 | 0.736 | 0.704 | 0.718 |

The AUC on the subsets of Dataset 1 are comparable to what we obtained in the main text (0.822) for the whole Dataset 1, except that of Dataset1_NoWork which is much higher. However, the AUC on the subsets of Dataset 2 are all much lower than 0.789. Hence, topic-specific models seemed to have triggered overfitting on the train sets and decreased rather than improved out-of-sample prediction performance.

# Robustness Check 4: Sensitivity to Train Set

One may wonder whether our results are influenced by the specific tweets in the train and test sets. To test this, we randomly split the union of Dataset 1 and Dataset 2 into a train set and a test set and replicated the analysis: run a MANOVA on the train set, select variables that were significant (if any), build a logit model (Forward, starting from the variables with highest absolute Cohen’s d) with the train set data and then using the model to predict Veracity in the test set. We repeated this process 1,000 times and report the AUC on test set in Figure S1.

In this robustness check, we only used the model obtained with the Forward approach, because it was not feasible to inspect 1,000 times what the other approaches gave on the train sets. It may be that, for some train sets, comparing the approaches would have led us to select another model. Hence, we can expect these brute-force simulations to produce slightly worse results than the step-by-step approach we took to build the personalized model.


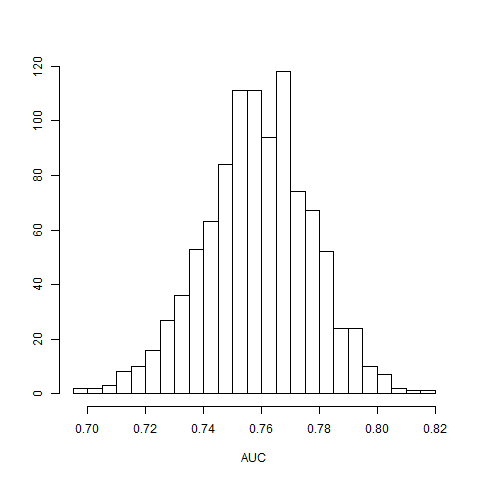


*Figure S1:* Histograms of AUC for 1,000 simulations varying the train set and the test set

The mean AUC over the 1,000 test sets is 0.759. The histogram in Figure S1 depicts the full distribution of AUC on the test sets. It shows that the test performance is relatively stable and that the AUC of 0.789 we obtained on Dataset 2 is not out-of-the ordinary. Reversing the training set and the test set with respect to the paper gives an AUC of 0.755, which is close to the simulation average.

# Robustness Check 5: Placebo Check

As a placebo check, we randomly shuffled the Veracity variables in Dataset 1 and in Dataset 2. We then replicated the analysis, exactly as in the previous robustness check, from the MANOVA to the out-of-sample prediction. Not surprisingly, the distribution of AUC is centered at 0.5, which means that it is not better than random. Comparing Figures S1 and S2, we can conclude that the AUC obtained in our linguistic analysis are extremely unlikely to be due to chance.


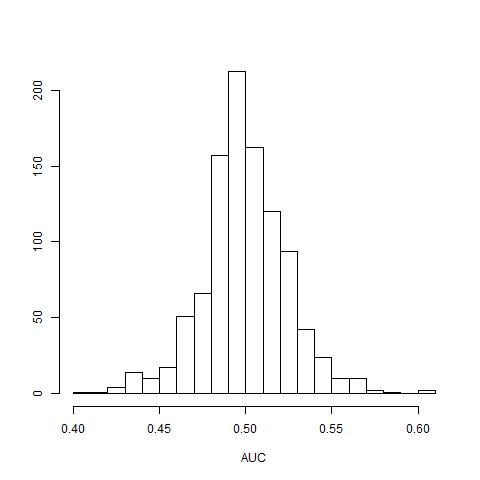


*Figure S2:* Histograms of AUC for the placebo check, shuffling the Veracity variable
